# The 9th Annual Scientific Meeting of the Asian Association for the Study of Diabetes (AASD) General Information

**DOI:** 10.1111/jdi.12683

**Published:** 2017-05-05

## President

Jiro Nakamura M.D., Ph.D.

Division of Diabetes, Department of Internal Medicine, Aichi Medical University School of Medicine, Japan

## Date

May 19 (Fri.) – 20 (Sat.), 2017

## Venue

Nagoya Congress Center

1‐1 Atsuta‐nishimachi, Atsuta‐ku, Nagoya 456‐0036, Japan

## Session Rooms

Plenary Sessions: Room 6 (Bldg.1, 4F, Reception Hall (west))

Oral Sessions: Room 5 (Bldg.1, 4F, Reception Hall (east))

Poster Sessions: Poster Room 1 (Underground Parking 4)

## Headquarter (During the Congress)

Nagoya Congress Center, Bldg. 4, Room 438

## Secretariat

Secretary General

Hideki Kamiya, M.D., Ph.D.

Associate Professor

Division of Diabetes,

Department of Internal Medicine,

Aichi Medical University School of Medicine

1‐1 Yazakokarimata, Nagakute, Aichi, 480‐1195, Japan

## Congress Secretariat

Japan Convention Services, Inc.

Kansai Regional Office

4‐4‐7 Imabashi, Chuo‐ku, Osaka, 541‐0042, Japan

TEL: +81‐6‐6221‐5933

E‐mail: 60jds@convention.co.jp

## Information for Participants

### Registration

**1) Registration Counter**

[Location]

Information Counter: Bldg.1, 1F, Event Hall

[Hours]

May 19 (Fri.) 07:00‐18:00

May 20 (Sat.) 07:00‐17:00

**2) Pre‐Registered Participants**

Please bring a registration confirmation letter to the information counter. The letter was sent to all participants who completed registration and payment in advance (until 12:00 on April 21).

**3) On‐site Registration**

Come to the information counter during the operating hour.

**4) Registration Fee**

**Table:** 

Category	Pre‐Registration (till 12:00 on April 21)	On‐Site
Regular	12,000 JPY	15,000 JPY
Student*1	Free	Free
Trainee*2	Free	Free

Payment must be made in Japanese yen with a credit card (Visa, MasterCard, JCB, American Express, and Diners Club) or by cash. No other method of payment is accepted.

^*1^Student participants are required to submit a copy of student ID and an official letter certifying their status.

^*2^All trainees are required to submit Certification of Training.

Registration fee includes:

- Admission to all scientific sessions of 9 AASD and the 60^th^ Japan Diabetes Society Annual Scientific Meeting (60 JDS);
- Access to Exhibits;
- Participation to any of the sponsored luncheon seminars of 9 AASD and 60 JDS with a lunch box; and
- Congress Kit, including, Program Book.

**5) Name Badge**

Name badge will be used as a pass. Badges must be worn at all times in Nagoya Congress Center, and other venues for 60 JDS. You will not be allowed to access to the Exhibit Hall or scientific sessions without a badge.

### Cloak

Facilities for luggage storage and coat check will be available during the congress. Each facility can store your bag(s) and coat(s) for the day.

Location: (1) Bldg.1, 1F, Atrium Lobby

                (2) 1F, Outside Corridor between Bldg.1 and Bldg.2

### Congress Kit

A congress kit can be picked up in exchange for a kit ticket. The ticket is attached to a name badge. Please be advised that without the ticket, you cannot pick up the kit.

Pick‐up Location: Bldg.1, 1F, Event Hall

### Sponsored Seminars

9 AASD and 60 JDS will have Luncheon and Evening Seminars. A ticket is required to join any of 9 AASD and 60 JDS seminars. Pre‐registered participants can reserve and receive a ticket in advance of the congress. On‐site registrants may pick up a ticket on the condition that the seats are available for the seminar of their choice.

Seminar Ticket Reservation Counter: Nagoya Congress Center, Bldg.2, 1F, Escalator Hall

Hours:   <Luncheon Seminar>

                   The day of a seminar, from 7:30 to 11:30

             <Evening Seminar>

                   The day of a seminar, from 13:00 to 18:00

                   * Tickets for 9 AASD Evening Seminar on 19 (Fri.) will not be available after 17:00.

### Exhibits

Location: (1) Bldg.1, 1F, Event Hall

                (2) Bldg.4, 1F, Shiratori Room

                (3) Bldg.2, 2F, Rooms 221, 225, 3F, Room 231

## Information for Chairs

### Symposium and Oral Presentations

Please take the next chairperson's seat in the room at least 20 minutes before the beginning of your session. Chairpersons are asked to remain within the time allotted for the session and each presentation.

### Poster Presentations

Please come to the Poster Chair Check‐In Counter in the poster room 1 (underground parking 4) at least 20 minutes before the beginning of your session.

## Information for Speakers

### Conflict of Interest (COI) Disclosure

Every presenter of 9 AASD is required to disclose COI status of their presentations. If the research included in a presentation was financially supported by any companies or other for‐profit groups, the role of the companies or the groups in analyzing the data or preparing the abstract must be clearly indicated. In oral presentations, COI status of the abstract must be shown in the 2nd slide (after the title slide). In poster presentations, it should be at the end of the poster. Samples of the disclosure slide are available on the JDS website: http://www.jds.or.jp/modules/about/index.php?content_id=13.

### Speakers of Symposium and Oral Sessions

**1) Time Allotment**

All speakers are kindly requested to strictly observe the allocated presentation time. All oral session rooms will be equipped with a Speaker Timing Device (a light‐signal system) in order to help speakers and chairs to keep within the allocated time limit for each presentation. A yellow light will flash 1 minute before the allocated presentation time, and a red light will flash at the end of the time.

1. Symposium and Award Lectures
  Allocated time will be announced separately.
2. Oral Sessions
  7 minutes talk and 3 minutes discussion.

**2) Presentation Style**

Accepted presentation style for 9^th^ AASD oral sessions is computer presentation. Speakers are recommended to bring their presentations on a USB flash drive or CD‐R. Alternatively, speakers may bring their own laptops. Mac users, however, should bring their own laptops, as the computers available onsite will only support a windows platform (OS: Windows 10). Whether bringing a USB / CD‐R or laptop, speakers are urged to bring a backup copy of their presentations as well.

**3) Speaker Check‐in**

Speakers should visit the PC Preview Center no later than one hour prior to their sessions in order to preview and upload the presentations, and for those bringing their laptops to check the compatibility of the laptops with AASD computers. Speakers of the first session of each day may come to and check‐in at the center the day before their presentations.

Location: Bldg. 1, 2F, Foyer of Century Hall

Hours:   May 18 (Thu.) 07:30‐18:30

              May 19 (Fri.) 07:30‐17:30

              May 20 (Sat.) 07:30‐16:00

**4) Presentation Using Speakers’ Own Laptops**

1. At the PC Preview Center

Before coming to the Center, please make it sure that your presentation will be displayed properly in 1024 x 768 resolution, and set the screensaver, power saving function, and password on your laptop to turn off. Connect your laptop to the preview monitor, using your own AC adapter and connecter, and check the configuration of your laptop with the monitor. An AASD congress staff will help you connect your laptop.

What to bring: Along with laptop, you must bring AC adapter and connecter. The connector available at the PC Preview Center is a D‐sub 15 pin (mini). Therefore, please bring in a D‐sub 15 pin female output and AC adapter. Mac users are also asked to bring a conversion connector.

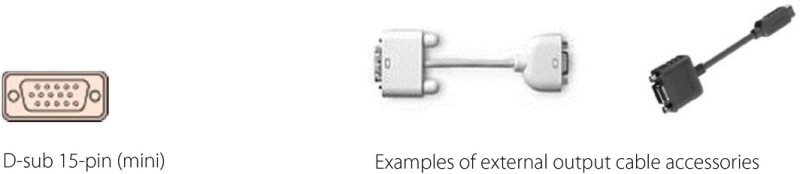

Recommended OS for preparing your presentations are Windows 7 and Mac OX 10.1.2 or later. If your presentation includes audio or video, you need to notify the AASD staff. Please have a backup copy, in case there is a problem with your original presentation stored in your laptop.

2. At the Session Room

Bring your laptop to the computer desk in front of the session room. Connecting your laptop with the AASD computer, an AV technician will make your presentation ready. Take the Next Speaker's Seat while the former presenter is speaking. The first page of your presentation will be displayed. Use a mouse or a pointer on the podium to advance to subsequent slides as you proceed with your presentation. Presenter View should not be used during the presentation. Pick up your laptop at the computer desk after your presentation.

**5) Presentation Using USB flash drive or CD‐R**

1. Saving Your Presentation

The only available presentation software in each session room will be PowerPoint 2010, 2013 and 2016. In order to avoid display problems, use only standard OS fonts such as Helvetica, Arial, and Times New Roman. When saving your presentation on a CD‐R, please use only the hybrid format (ISO9660), as special functions such as “Packet Write” may make your data illegible.

2. Data Management

The resolution of the session room screen is XGA (1024 x 768). Maximum 512 MB is allowed for oral presentations. You can use audio or video in your presentation. We recommend any video data to be in WMV format which can be played with Windows Media Player 11. All data files should be in one folder, including any reference files such as video images. Please have a backup copy of your data ready, in case there is any problem with your original presentation. To avoid the possible spread of computer viruses, always scan your presentation files beforehand with updated anti‐virus software. After saving your presentation file on the appropriate medium, run a test on another computer to make sure it works normally.

3. At the PC Preview Center

Please confirm that your presentation is displayed properly on the preview monitor. If your presentation includes audio or video, please notify the AASD staff. The staff will help you to register your presentation. Registered presentation will then be downloaded to your session room.

4. At the Session Room

Take the Next Speaker's Seat while the former presenter is speaking. The first page of your presentation will be displayed. Use a mouse or a pointer on the podium to advance to subsequent slides as you proceed with your presentation. Presenter View should not be used during the presentation. Your presentation will be properly erased from all the computers of the AASD rooms.

## Speakers of Poster Presentation

### Presenter Check‐in

Come to the Poster Presentation Check‐in Desk at latest 15 min. prior to your presentation. After check‐in, mount your poster materials on the numbered poster board assigned to your presentation.

### Poster Display Requirement

The poster board surface area is 90 cm width x 180 cm height. At the upper left of the board will be displayed a Poster No., which will be provided by AASD. Pushpins will be provided as well. Presenters are required to bring other items: i.e., Abstract Title, Organization(s) of the Author(s), Author(s) Name(s), and Presentation Materials.

### COI Disclosure Statement

All presenters are required to display COI disclosure statement in the last part of their poster materials.

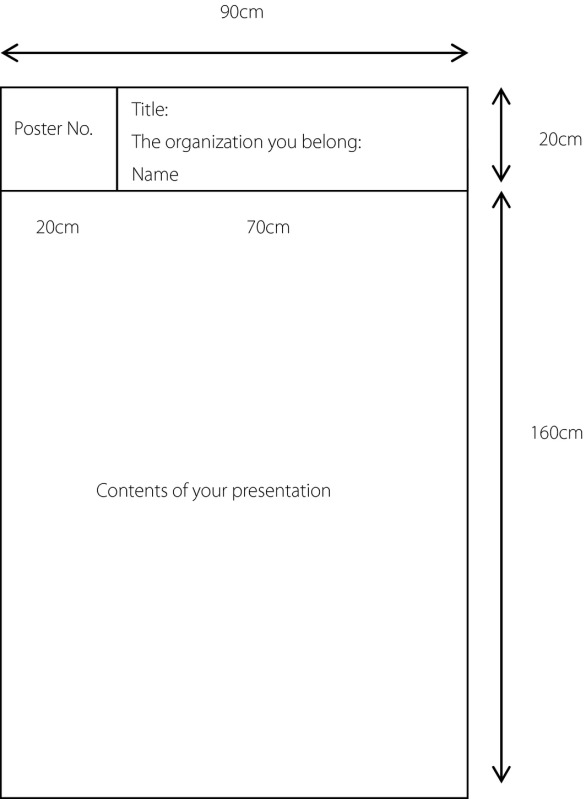

### Poster Session Schedule

Location: Poster Room 1 (Underground Parking 4)

Poster viewing, display, and session hours are listed below. At the session hour, each presenter is assigned 3 min. for their talk and 2 min. for discussion.

**Table:** 

	Mounting	Viewing	Poster Sessions	Tear‐Down
19 (Fri.)	8:00‐10:00	10:00‐17:40	17:40‐18:40	18:40‐19:00
20 (Sat.)	8:00‐10:00	10:00‐16:20	16:20‐17:20	17:20‐17:50

Please mount your presentation materials to the poster board with your number.

Presenters are expected to stand at their poster board during the assigned Poster Session time.

Do not leave your belongings, poster containers or any materials under the poster boards or in the poster area. AASD is not responsible for articles left in the poster area. The congress management staff will remove and discard posters that remain after the removal time. No poster removed by the staff will be kept for later collection.

